# Study on the Relationships Between Calcium Accumulation in Fruits and Calcium Dynamics in Fruit Stalks During the Development and Ripening of *Cerasus humilis* Fruit

**DOI:** 10.3390/plants14050655

**Published:** 2025-02-21

**Authors:** Li Zhang, Wei Zhang, Jinli Guo

**Affiliations:** College of Horticulture and Plant Protection, Inner Mongolia Agricultural University, Hohhot 010010, China; zlkx628@163.com (L.Z.); zhangwei980319@163.com (W.Z.)

**Keywords:** *Cerasus humilis*, fruit stalks, fruits, 2,4-D, calcium

## Abstract

*Cerasus humilis* is a unique fruit tree renowned for its exceptional calcium content. To investigate the relationship between calcium absorption and accumulation in the fruit, as well as the dynamic changes in calcium within the fruit stalks during development and maturation, this study examined the content and proportion of different forms of calcium in the fruits and fruit stalks under different *Cerasus humilis* types and 2,4-D treatment. Additionally, the characteristics of the calcium concentration difference ratio between the fruit stalks and fruits were analyzed. Given that the study is based on a single year of data, this preliminary research explores the calcium transport relationship between the fruit stalks and fruits, providing a foundation for more extensive future research. The results revealed that the content and proportion of water-soluble calcium in fruits and fruit stalks gradually increased during development. In contrast, the content and proportion of other calcium forms, such as calcium oxalate and calcium phosphate, showed a downward trend. This decline was more pronounced in the fruits than in the fruit stalks. Treatment with 2,4-D significantly increased the content of all calcium components in the fruits, reduced the proportion of calcium oxalate and calcium phosphate in the fruit stalks, and increased their proportion in the fruits. The changes in calcium forms between the fruits and fruit stalks were closely interrelated, with 2,4-D treatment enhancing the correlation between the contents of calcium phosphate and calcium oxalate in both tissues. The calcium concentration in the fruit stalks is significantly higher than in the fruits, showing a noticeable calcium concentration difference ratio, with water-soluble calcium exhibiting the smallest difference and calcium oxalate and calcium phosphate displaying the largest. Treatment with 2,4-D significantly reduced the calcium concentration difference ratio between the fruit stalks and the fruits. In conclusion, the periods from the young fruit stage to the hard seed stage and from the hard–ripe stage to the fully ripe stage are critical for calcium transformation, transport, and accumulation. Different calcium forms in the fruits and fruit stalks can interconvert, with water-soluble calcium being more readily transported to the fruits. In contrast, calcium oxalate and calcium phosphate tend to accumulate in the fruit stalks. Calcium pectin behaves differently in the two types of *Cerasus humilis*. In MY-2, it is more readily transported to the fruit, while in MY-9, it tends to accumulate in the fruit stalks. Treatment with 2,4-D facilitates the transport of calcium from the fruit stalks to the fruits, enhances calcium content in the fruits, and optimizes calcium distribution.

## 1. Introduction

*Cerasus humilis* (Bge.) Sok., a member of the subgenus *Microcerasus* in the *Rosaceae* family, is a unique fruit tree species endemic to China [1]. It is primarily found in northern regions such as Shanxi, Inner Mongolia, Liaoning, and Hebei. Known for its remarkable tolerance to drought, cold, and poor soil conditions, *Cerasus humilis* is highly effective in windbreaks and soil erosion control [2]. Its fruits, with vibrant coloration, unique flavor, and richness in mineral elements [3,4], are particularly notable for their high calcium content—ranging from 2 to 10 times that of other fruits—and their high bioavailability to humans, earning them the nickname ‘calcium fruit’ [5]. Additionally, the kernels of *Cerasus humilis* possess medicinal properties [6], and its leaves can be used to produce tea [7]. As a multi-functional plant with nutritional, medicinal, and economic value, *Cerasus humilis* holds significant potential for development in the food and health industries [8,9].

Calcium plays a crucial role in regulating metabolic processes within plants, participating in signal transduction, maintaining cell wall strength, and protecting the integrity of the cell membrane [10,11]. In *Cerasus humilis* fruits, calcium primarily exists in four different calcium components: water-soluble calcium, calcium pectin, calcium phosphate, and calcium oxalate. Among these, water-soluble calcium and calcium pectin are considered active calcium, which is beneficial for human absorption and utilization [12]. Different forms of calcium have distinct physiological functions, and under certain conditions, these calcium forms can interconvert [13]. In fruits, calcium is not only a key nutritional component but also essential for extending shelf life, improving storage properties, and enhancing market value [14,15,16]. As terminal organs with limited transpiration, fruits rely on the fruit stalk to transport nutrients, including calcium, from the plant body [17]. Understanding the pathways of calcium transport into fruits has been a central focus of calcium nutrition research [18]. The fruit stalk, serving as the bridge between the tree and the fruit, is crucial for calcium uptake and accumulation in the fruits, making it a key focus of calcium transport research [19,20]. Exogenous applications of auxins are known to promote fruit growth and development while enhancing calcium uptake. Auxins, such as indole-3-acetic acid (IAA), can increase cytoplasmic calcium concentrations and facilitate calcium transport from the plant body to the fruit [21]. Synthetic auxins, such as 2,4-D, have been shown to promote cell growth and differentiation, accelerate root and shoot elongation, and facilitate fruit formation and seed germination [22,23,24]. Studies have demonstrated that exogenous applications of IAA, gibberellic acid (GA), and naphthaleneacetic acid (NAA) enhance calcium transport into fruits. Notably, IAA has been reported to facilitate calcium translocation from the tree to the fruit and increase the activity of Ca^2+^-ATPase in the plasmalemma of apple fruit flesh [25,26], thereby promoting calcium ion absorption.

As a fruit species renowned for its exceptional calcium content, *Cerasus humilis* serves as an ideal model for studying the mechanisms of calcium uptake and accumulation. Understanding these processes could provide a foundation for improving fruit quality and supporting future resource innovation. Current research on *Cerasus humilis* calcium primarily focuses on calcium content and composition at different growth stages [27], as well as calcium changes under various storage conditions [28]. However, the relationship between calcium accumulation in fruits and its transport through the fruit stalk remains poorly understood. This study initially uses two types of *Cerasus humilis*, ‘Mengyou No. 2’ (MY−2) and ‘Mengyou No. 9’ (MY−9), as materials to systematically compare the changes in different calcium forms in the fruits and fruit stalks during fruit development and maturation. The relationship between calcium accumulation in the fruits and its transport through the fruit stalk is explored to uncover the mechanisms underlying calcium supply to the fruit. Additionally, this study examines the effects of the exogenous plant growth regulator 2,4-D on calcium dynamics in fruit stalks and fruits during development. By elucidating the role of 2,4-D in regulating calcium transport and accumulation, this research aims to determine whether 2,4-D can enhance fruit calcium supply by modulating calcium transport in the fruit stalks. The findings are expected to provide valuable insights into strategies for regulating fruit calcium content and advancing the application of plant growth regulators in fruit production systems.

## 2. Results

### 2.1. Changes in the Different Forms of Calcium in the Fruits and Fruit Stalks During the Development of Different Types of Cerasus humilis

#### 2.1.1. Changes in the Different Forms of Calcium in the Fruits of Different Types of *Cerasus humilis*

**Figure 1:**
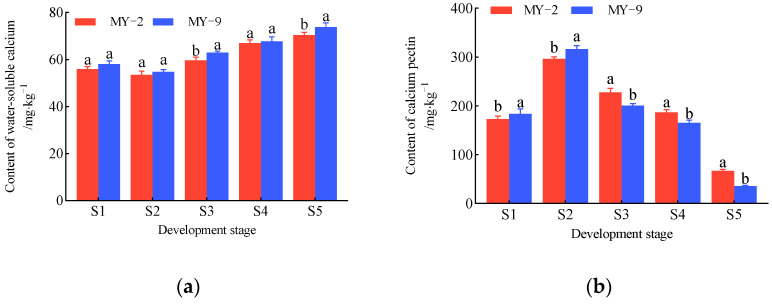

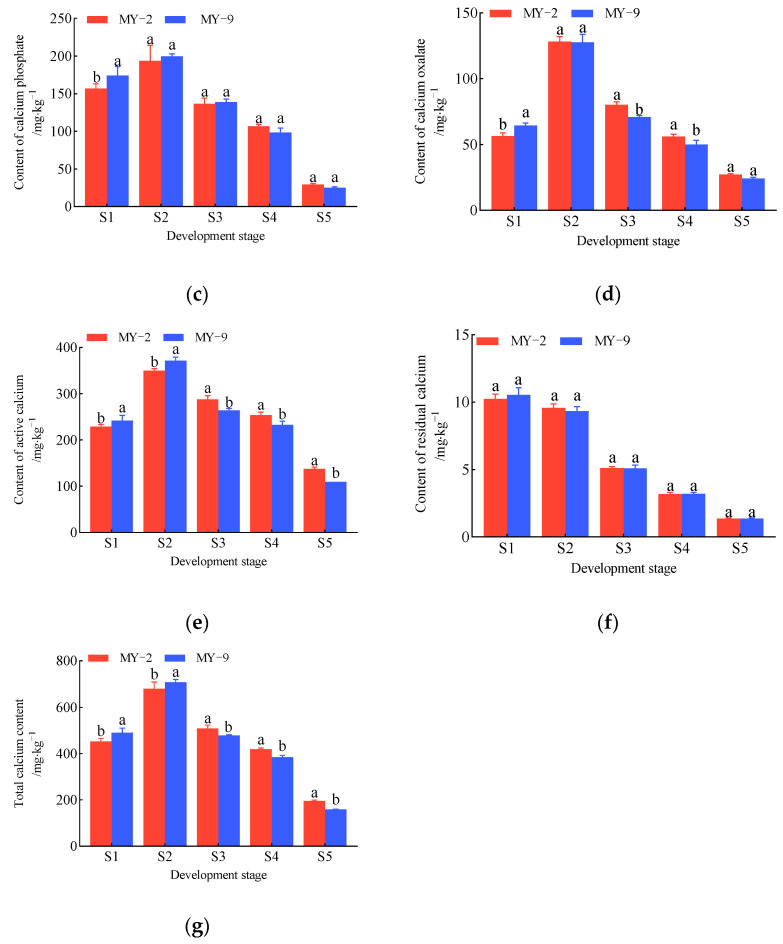
The change in calcium content of different forms during the development and ripening of *Cerasus humilis* in fruit: (**a**) is the content of water-soluble calcium; (**b**) is the content of calcium pectin; (**c**) is the content of calcium phosphate; (**d**) is the content of calcium oxalate; (**e**) is the content of active calcium; (**f**) is the content of residual calcium; and (**g**) is the content of total calcium. S1: young fruit stage; S2: hard seed stage; S3: coloring and enlargement stage; S4: hard–ripe stage; S5: fully ripe stage. Note: the lowercase letters indicate Duncan’s test results for *p* < 0.05 in the fruits of *Cerasus humilis* at different developmental stages for MY−2 and MY−9; the error bars represent the average standard deviation (*n* = 3).

During the development and ripening of *Cerasus humilis* fruits, the changes in various calcium forms in the MY-2 and MY-9 fruits are illustrated in Figure 1. In both types of *Cerasus humilis,* the water-soluble calcium content initially decreases before gradually increasing, with the lowest point observed at the hard seed stage. After this stage, the water-soluble calcium content accumulated rapidly, peaking at the fully ripe stage, thus displaying an overall increasing trend. Throughout fruit development, the water-soluble calcium content in MY-9 fruits remained consistently higher than in MY-2 fruits (Figure 1a). The levels of calcium pectin, calcium phosphate, calcium oxalate, active calcium, and total calcium followed a similar pattern, initially increasing and then gradually decreasing. These calcium forms peaked at the hard seed stage, followed by a sharp decline as the fruit matured, reaching their lowest levels at the fully ripe stage, exhibiting an overall downward trend (Figure 1b–e,g). In general, the levels of these calcium forms were higher in MY-9 fruits during the young fruit and hard seed stages. However, from the coloring and enlargement stage to the fully ripe stage, MY-2 fruits exhibited higher concentrations of these calcium forms compared to MY-9 fruits. The residual calcium content in both fruit types consistently decreased throughout development, with only trace amounts remaining by the fully ripe stage (Figure 1f).

In conclusion, while the patterns of changes in calcium forms during fruit development and maturation are similar between the two types of *Cerasus humilis*, there are differences in calcium levels. At the fully ripe stage, MY-9 fruit exhibits higher water-soluble calcium content, while MY-2 fruit shows higher levels of the other calcium forms. Regarding the trends for each calcium form, calcium pectin, calcium phosphate, calcium oxalate, active calcium, and total calcium all display consistent downward trends. In contrast, water-soluble calcium exhibited an opposite trend, steadily increasing as the fruit matured (Figure 1).

#### 2.1.2. Changes in the Different Forms of Calcium in the Fruit Stalks of Different Types of *Cerasus humilis*

**Figure 2:**
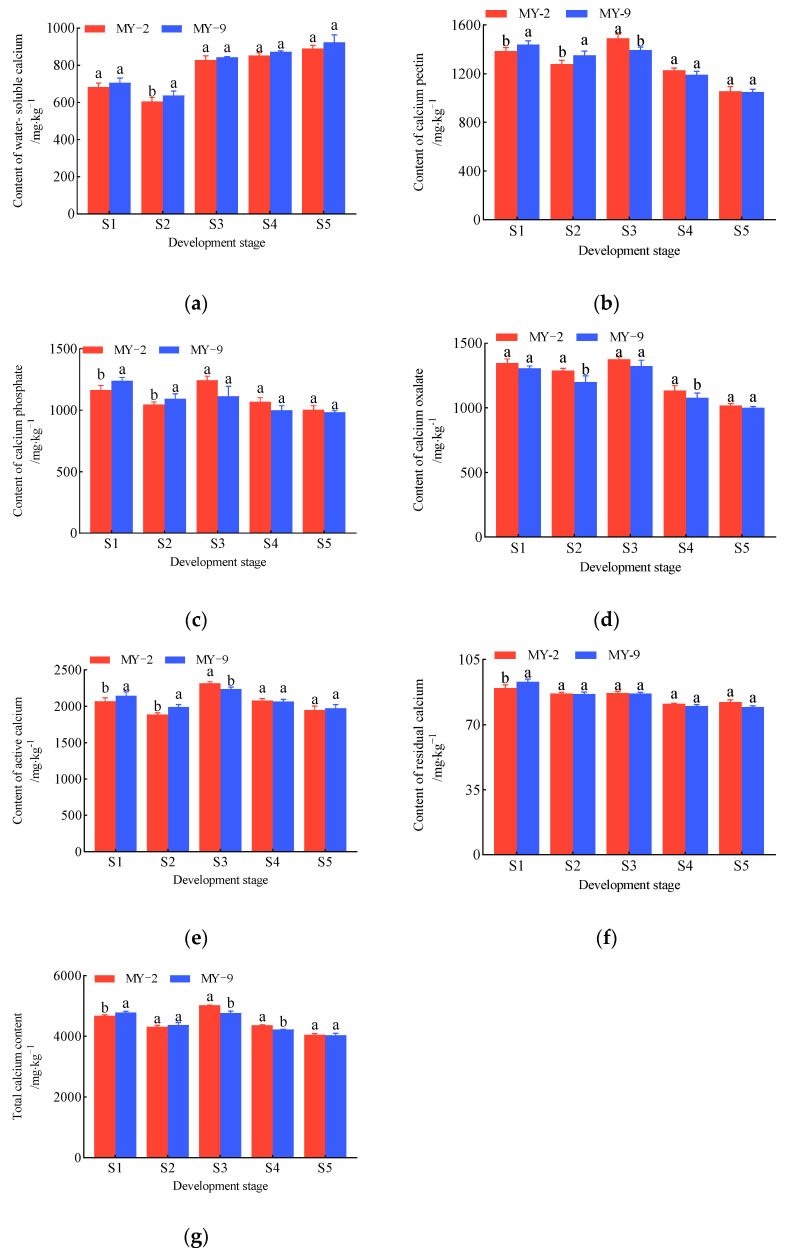
The change in calcium content of different forms during the development and ripening of *Cerasus humilis* in fruit stalk: (**a**) is the content of water-soluble calcium; (**b**) is the content of calcium pectin; (**c**) is the content of calcium phosphate; (**d**) is the content of calcium oxalate; (**e**) is the content of active calcium; (**f**) is the content of residual calcium; and (**g**) is the content of total calcium. S1: young fruit stage; S2: hard seed stage; S3: coloring and enlargement stage; S4: hard–ripe stage; S5: fully ripe stage. Note: the lowercase letters indicate Duncan’s test results for *p* < 0.05 in the fruit stalks of *Cerasus humilis* at different developmental stages for MY−2 and MY−9; the error bars represent the average standard deviation (*n* = 3).

During the development and ripening of *Cerasus humilis* fruits, the dynamics of various calcium forms in the fruit stalks of MY-2 and MY-9 *Cerasus humilis* are depicted in Figure 2. In both types of *Cerasus humilis*, the water-soluble calcium content in the fruit stalks reached its lowest point at the hard seed stage. After this stage, the water-soluble calcium content began to accumulate rapidly, peaking at the fully ripe stage and displaying an overall increasing trend. Throughout the entire developmental process, the water-soluble calcium content in the fruit stalks of MY-9 was consistently slightly higher than that of MY-2 (Figure 2a). The contents of calcium pectin, calcium phosphate, calcium oxalate, active calcium, and total calcium in the fruit stalks followed similar patterns: they peaked during the coloring and enlargement stage and gradually declined thereafter, reflecting an overall downward trend. Before the hard seed stage, the levels of these five calcium forms were generally higher in MY-9 fruit stalks than in MY-2 fruit stalks. However, from the hard seed stage to the fully ripe stage, the trend reversed, with higher calcium contents observed in MY-2 fruit stalks compared to MY-9 fruit stalks (Figure 2b–e,g). The residual calcium content in the fruit stalks was highest during the young fruit stage and decreased progressively as the fruit developed and matured (Figure 2f).

In summary, despite similar patterns in the changes of calcium forms in the fruit stalks during fruit development and maturation, there are differences in the calcium levels between the two *Cerasus humilis* types. The levels of calcium pectin, calcium phosphate, calcium oxalate, active calcium, total calcium, and residual calcium followed largely consistent patterns, generally declining over time. In contrast, the water-soluble calcium content showed an opposite trend, steadily increasing throughout fruit development (Figure 2).

The variation patterns of calcium forms in the fruits and fruit stalks of the two *Cerasus humilis* types (MY-2 and MY-9) are summarized in Figure 1 and Figure 2. The trends in calcium forms in both the fruits and the fruit stalks were similar, with an overall increase in water-soluble calcium and decreases in total calcium, active calcium, calcium pectin, calcium phosphate, calcium oxalate, and residual calcium. At the fully ripe stage, compared to the young fruit stage, the water-soluble calcium content in MY-2 and MY-9 fruits increased by 25.93% and 27.02%, respectively (Figure 1a). In the fruit stalks, the increases were 30.41% and 30.94%, respectively (Figure 2a). In the MY-2 fruits, the contents of total calcium, active calcium, calcium pectin, calcium phosphate, and calcium oxalate decreased by 56.88%, 40.05%, 61.35%, 81.28%, and 51.83%, respectively (Figure 1b–e,g). In the MY-2 fruit stalks, these forms of calcium decreased by 13.27%, 5.95%, 23.83%, 13.79%, and 24.38%, respectively (Figure 2b–e,g). In the MY-9 fruits, the reductions in total calcium, active calcium, calcium pectin, calcium phosphate, and calcium oxalate were 67.42%, 54.84%, 80.69%, 85.58%, and 62.39%, respectively (Figure 1b–e,g). In the MY-9 fruit stalks, these calcium forms decreased by 15.65%, 8.04%, 27.13%, 20.65%, and 23.46%, respectively (Figure 1b–e,g). These results show that the increase in water-soluble calcium in the fruit was slightly smaller than in the fruit stalk, while the decreases in total calcium, active calcium, calcium pectin, calcium phosphate, and calcium oxalate were significantly greater in the fruits than in the fruit stalks. Compared to MY-2, MY-9 exhibited a greater change in both the increase and decrease in calcium forms in the fruits and fruit stalks (Figure 1 and Figure 2).

### 2.2. Changes in the Different Forms of Calcium in the Fruits and Fruit Stalks of Cerasus humilis Under 2,4-D Treatment

#### 2.2.1. Changes in the Different Forms of Calcium in the Fruits of *Cerasus humilis* Under 2,4-D Treatment

**Figure 3:**
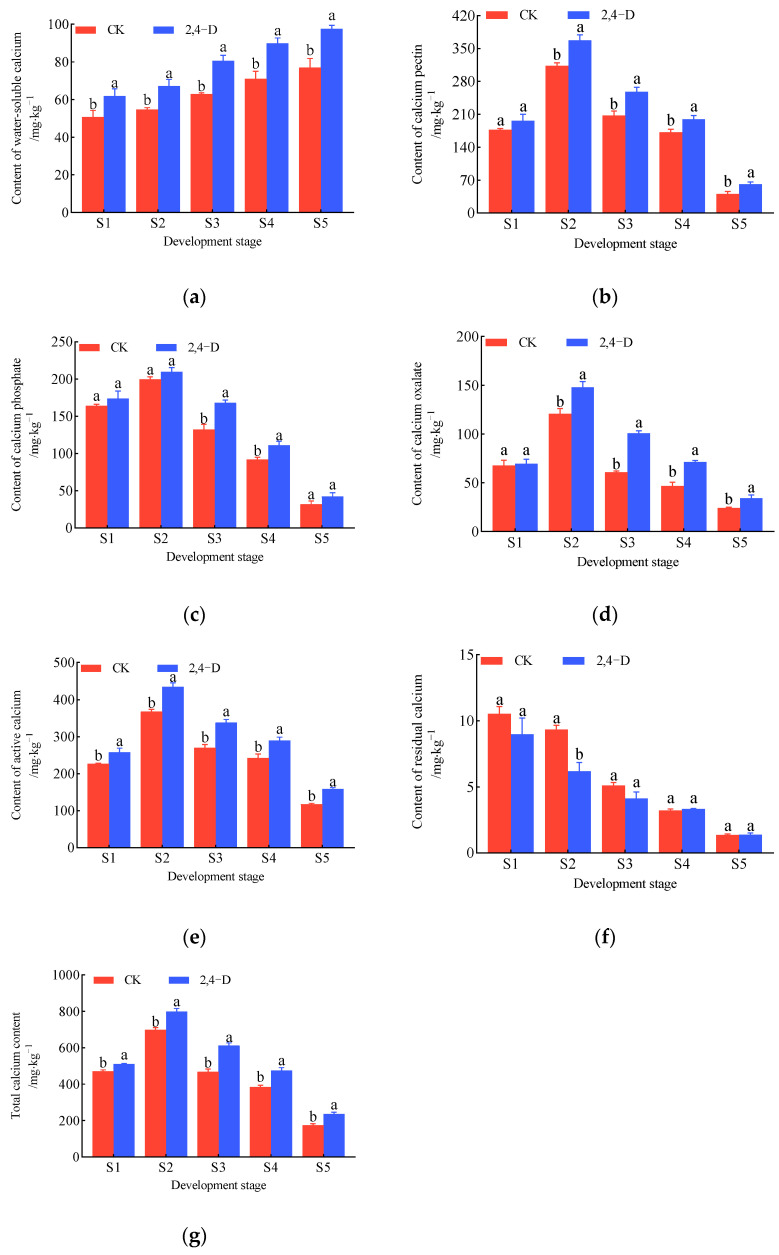
The change in calcium content of different forms during the development and ripening of *Cerasus humilis* in fruit under 2,4-D treatment: (**a**) is the content of water-soluble calcium; (**b**) is the content of calcium pectin; (**c**) is the content of calcium phosphate; (**d**) is the content of calcium oxalate; (**e**) is the content of active calcium; (**f**) is the content of residual calcium; and (**g**) is the content of total calcium. S1: young fruit stage; S2: hard seed stage; S3: coloring and enlargement stage; S4: hard–ripe stage; S5: fully ripe stage. Note: the lowercase letters indicate Duncan’s test results for *p* < 0.05 in the fruits of *Cerasus humilis* at different developmental stages for CK (distilled water spraying was used as the control and labeled as CK) and 2,4-D; the error bars represent the average standard deviation (*n* = 3).

During the development and maturation of *Cerasus humilis*, the changes in the content of different calcium forms in the fruits under 2,4-D treatment and control (distilled water spraying) are shown in Figure 3. The water-soluble calcium content in the fruits showed a gradual increase, reaching its highest level at the fully ripe stage. Throughout fruit development, the water-soluble calcium content in the 2,4-D-treated fruits was significantly higher than that in the control (Figure 3a). The contents of calcium pectin, calcium phosphate, calcium oxalate, active calcium, and total calcium in the fruits exhibited an overall increase followed by a decrease, peaking at the hard seed stage and gradually declining as the fruit matured, with the lowest content at the fully ripe stage. Throughout all developmental stages, the contents of these five calcium forms were consistently higher in the 2,4-D-treated fruits compared to the control (Figure 3b–e,g). The residual calcium content in the fruits followed a gradual decreasing trend, with a noticeable reduction in overall content (Figure 3f).

In summary, the trends in the changes of different calcium forms in the 2,4-D-treated and control fruits were generally similar, but the content differences were substantial. The application of 2,4-D significantly increased the content of various calcium forms in *Cerasus humilis* fruits (Figure 3).

#### 2.2.2. Changes in the Different Forms of Calcium in the Fruit Stalks of *Cerasus humilis* Under 2,4-D Treatment

**Figure 4:**
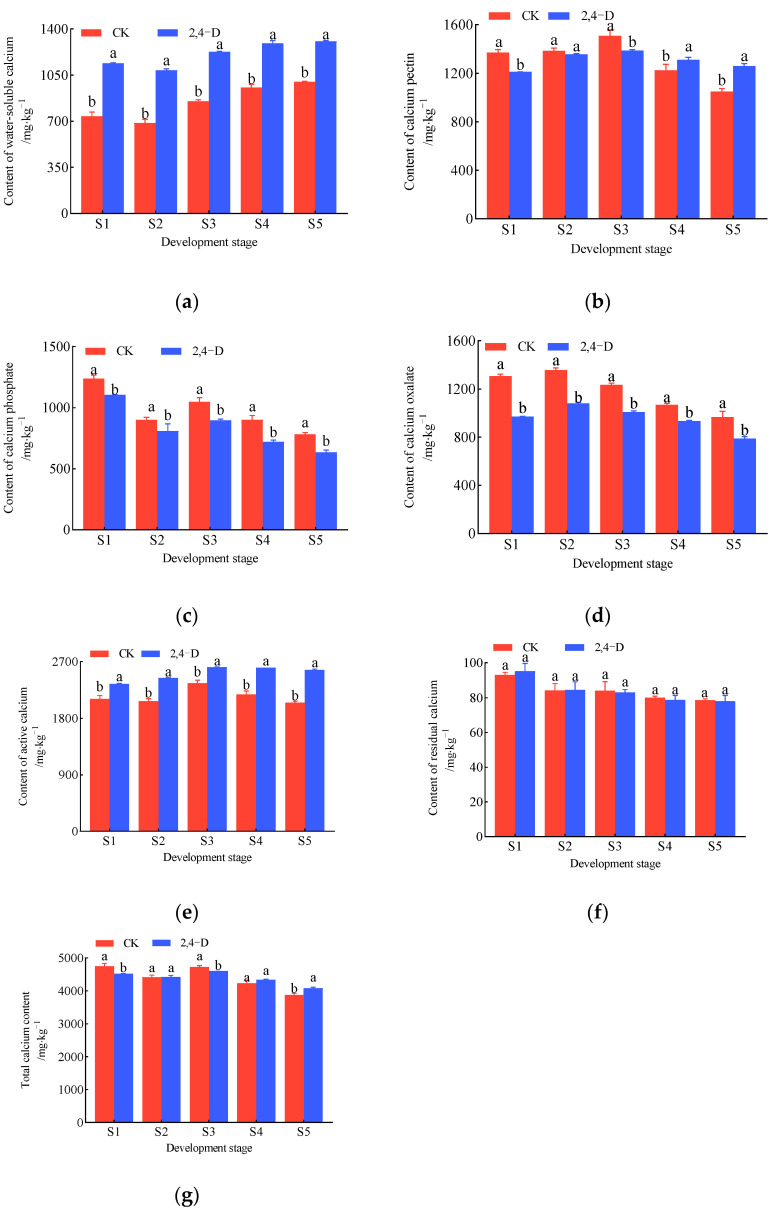
The change in calcium content of different forms during the development and ripening of *Cerasus humilis* in fruit stalk under 2,4-D treatment: (**a**) is the content of water-soluble calcium; (**b**) is the content of calcium pectin; (**c**) is the content of calcium phosphate; (**d**) is the content of calcium oxalate; (**e**) is the content of active calcium; (**f**) is the content of residual calcium; and (**g**) is the content of total calcium. S1: young fruit stage; S2: hard seed stage; S3: coloring and enlargement stage; S4: hard–ripe stage; S5: fully ripe stage. Note: the lowercase letters indicate Duncan’s test results for *p* < 0.05 in the fruit stalk of *Cerasus humilis* at different developmental stages for CK (distilled water spraying was used as the control and labeled as CK) and 2,4-D treatment; the error bars represent the average standard deviation (*n* = 3).

During the development and maturation of *Cerasus humilis* fruits, the changes in the content of different calcium forms in the fruit stalks under 2,4-D treatment and the control group (distilled water spraying) are shown in Figure 4. The water-soluble calcium content exhibited an overall increasing trend, with the lowest content at the hard seed stage and the highest at the fully ripe stage. Throughout the development, the water-soluble calcium content in the 2,4-D-treated fruit stalks was significantly higher than in the control (Figure 4a). The calcium pectin content in the fruit stalks exhibited an initial increase followed by a subsequent decrease under both 2,4-D treatment and control conditions. It peaked during the coloring and enlargement stage and reached its lowest level at the fully ripe stage. During the early stages of fruit development (from the young fruit stage to the coloring and enlargement stage), the calcium pectin content in the 2,4-D-treated fruit stalks was lower than that in the control. However, during the fruit maturation stage, the calcium pectin content in the 2,4-D-treated fruit stalks was significantly higher than in the control (Figure 4b). The changes in calcium phosphate and calcium oxalate contents in the fruit stalks followed similar trends under both conditions, showing an overall decreasing pattern, with the lowest content at the fully ripe stage. Throughout fruit development, the calcium phosphate and calcium oxalate contents in the 2,4-D-treated fruit stalks were significantly lower than in the control (Figure 4c,d). The active calcium content in the fruit stalks displayed an initial increase followed by a decrease, peaking during the coloring and enlargement stage. Throughout fruit development, the active calcium content in the 2,4-D-treated fruit stalks was significantly higher than in the control (Figure 4e). The residual calcium content in the fruit stalks exhibited a general decreasing trend, with minimal differences between treatments (Figure 4f). The total calcium content in the fruit stalks showed an overall decreasing trend, with the 2,4-D-treated fruit stalks having lower total calcium content than the control from the young fruit stage to the coloring and enlargement stage, but higher total calcium content at the fully ripe stage (Figure 4g).

These results indicate that during fruit development and maturation, the patterns of change in calcium content in the fruit stalks under 2,4-D treatment and the control are similar, but there are differences in content. The 2,4-D treatment increases the contents of water-soluble calcium and active calcium in the fruit stalks throughout development, while reducing the contents of calcium phosphate and calcium oxalate. The treatment has a reducing effect on the calcium pectin and total calcium contents in the fruit stalks during the early stages of fruit development but increases these contents at the fully ripe stage (Figure 4).

Integrating the changes in different calcium forms in the fruits and fruit stalks under 2,4-D treatment (Figure 3 and Figure 4), both the 2,4-D-treated and control groups exhibited an increasing trend in water-soluble calcium in the fruits and fruit stalks, while the contents of calcium pectin, calcium phosphate, calcium oxalate, residual calcium, and total calcium showed decreasing trends. Compared to the young fruit stage, at the fully ripe stage, the water-soluble calcium content in the 2,4-D-treated fruits increased by 57.61%, compared to 51.97% in the control (Figure 3a). The calcium pectin content decreased by 68.71% in the 2,4-D-treated fruits and 76.95% in the control (Figure 3b), calcium phosphate decreased by 75.52% in the 2,4-D-treated fruits and 80.63% in the control (Figure 3c), calcium oxalate decreased by 50.84% in the 2,4-D-treated fruits and 64.24% in the control (Figure 3d), and total calcium decreased by 53.56% in the 2,4-D-treated fruits and 62.73% in the control (Figure 3g). These results suggest that 2,4-D treatment enhanced the increase in water-soluble calcium in the fruit while reducing the decline in calcium pectin, calcium phosphate, calcium oxalate, and total calcium. Compared to the young fruit stage, at the fully ripe stage, the water-soluble calcium content in the 2,4-D-treated fruit stalks increased by 14.85%, compared to 35.49% in the control (Figure 4a). Calcium phosphate decreased by 42.59% in the 2,4-D-treated fruit stalks and 36.79% in the control (Figure 4b), calcium oxalate decreased by 18.66% in the 2,4-D-treated fruit stalks and 26.01% in the control (Figure 4c), and total calcium decreased by 9.76% in the 2,4-D-treated fruit stalks and 18.32% in the control (Figure 4g). These results indicate that 2,4-D treatment reduced the decline in calcium oxalate and total calcium in the fruit stalks but increased the reduction in calcium phosphate.

### 2.3. Changes in the Proportional Distribution of Different Forms of Calcium in the Fruits and Fruit Stalks During the Development of Cerasus humilis

#### 2.3.1. Changes in the Proportional Distribution of Different Calcium Forms in the Fruits and Fruits Stalks of Different Types of *Cerasus humilis*

**Figure 5:**
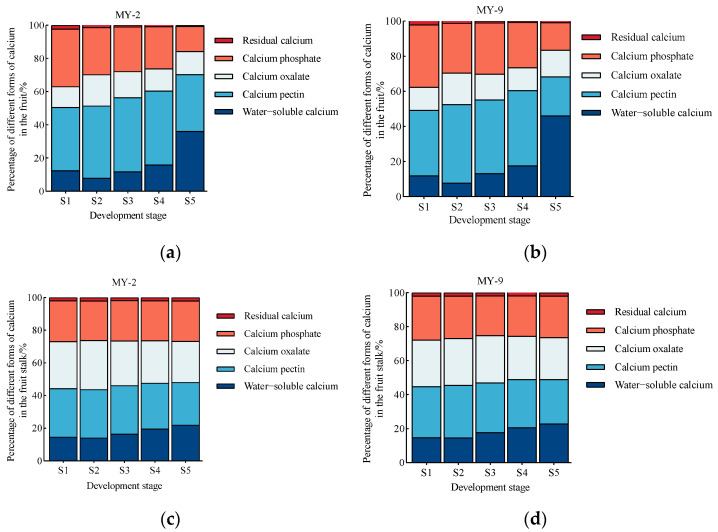
Changes in the proportions of calcium components during the development and maturation of *Cerasus humilis* in the fruit and fruit stalk: (**a**) is the percentage of calcium components in *Cerasus humilis* MY−2 fruit; (**b**) is the percentage of calcium components in *Cerasus humilis* MY−9 fruit; (**c**) is the percentage of calcium components in *Cerasus humilis* MY-2 fruit stalk, and (**d**) is the percentage of calcium components in *Cerasus humilis* MY-9 fruit stalk. S1: young fruit stage; S2: hard seed stage; S3: coloring and enlargement stage; S4: hard–ripe stage; S5: fully ripe stage.

During the development and maturation of *Cerasus humilis,* the changes in the proportions of different calcium forms in the fruits and fruit stalks of the MY-2 and MY-9 types are shown in Figure 5. In both types, the proportion of water-soluble calcium in the fruits and fruit stalks exhibited an overall increasing trend, with the highest proportion at the fully ripe stage. In the fruits, the proportion exceeded 35%, and in the fruit stalks, it exceeded 20%. The proportion of calcium pectin showed a decreasing trend, reaching the lowest proportion at the fully ripe stage. The proportion of active calcium, defined as the sum of water-soluble calcium and calcium pectin, exhibited an increasing trend in the fruits and fruit stalks, peaking at the fully ripe stage. In the fruits, the proportion of active calcium approached 70%, making it the predominant calcium form, while in the fruit stalks, the proportion of active calcium exceeded 48%. The proportion of calcium oxalate exhibited a trend of first increasing and then decreasing, peaking at the hard seed stage. The proportion of calcium phosphate decreased gradually in the fruits, while the trend in the fruit stalks was less pronounced. At the fully ripe stage, the proportions of the four major calcium components in the fruits of the two types differed significantly, following this order: water-soluble calcium > calcium pectin > calcium phosphate > calcium oxalate (Figure 5a,b). In the fruit stalks, the proportions of the four major calcium components were more similar, with the order being calcium pectin > calcium oxalate > calcium phosphate > water-soluble calcium (Figure 5c,d). Compared to MY-2 (Figure 5a,c), MY-9 fruits and fruit stalks showed higher proportions of water-soluble calcium, while the proportions of calcium pectin, calcium phosphate, and calcium oxalate proportions were lower.

In summary, at the fully ripe stage, compared to the fruit stalks, both the MY-2 and MY-9 fruits exhibited significantly higher proportions of active calcium and water-soluble calcium. In the MY-2 fruits, the proportion of calcium pectin was significantly higher than in the fruit stalks, while in the MY-9 fruits, the proportion of calcium pectin was significantly lower than in the fruit stalks. The proportions of calcium phosphate and calcium oxalate were lower in the fruits compared to the fruit stalks, suggesting that MY-2 fruit stalks tend to retain more calcium phosphate and calcium oxalate, while water-soluble calcium and calcium pectin are more easily transported to the fruit. In contrast, in MY-9 fruit stalks, water-soluble calcium is easily transported to the fruit, while calcium phosphate, calcium oxalate, and calcium pectin are more likely to remain in the fruit stalks. Throughout fruit development and maturation, the proportion of water-soluble calcium in the fruit stalks continually increased, while the proportions of other calcium forms remained relatively stable with minor fluctuations. In the fruits, the proportions of different calcium forms showed clear differences between the young fruit and the fully ripe stages, with the most significant changes occurring during the late fruit development stages, particularly between the hard–ripe stage and the fully ripe stage. These two periods are critical for calcium changes, transport, and accumulation (Figure 5).

#### 2.3.2. Changes in the Proportional Distribution of Different Calcium Forms in the Fruits and Fruits Stalks of *Cerasus humilis* Under 2,4-D Treatment

**Figure 6:**
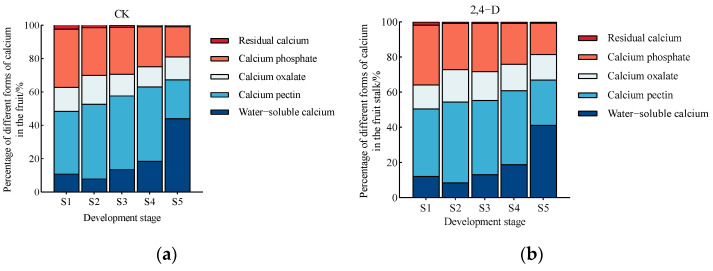

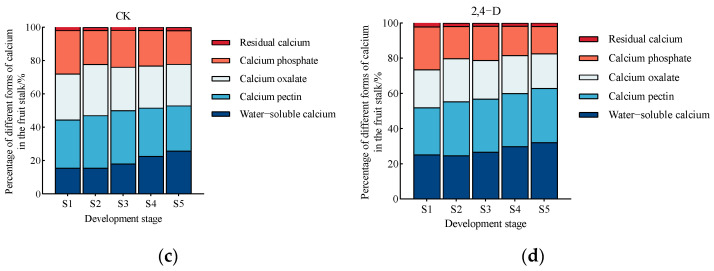
Changes in the proportions of calcium components during the development and maturation of *Cerasus humilis* in the fruit and fruit stalk under 2,4-D treatment: (**a**) is the percentage of calcium components in CK *Cerasus humilis* fruit; (**b**) is the percentage of calcium components in 2,4-D treatment *Cerasus humilis* fruit; (**c**) is the percentage of calcium components in CK *Cerasus humilis* fruit stalk; and (**d**) is the percentage of calcium components in 2,4-D treatment *Cerasus humilis* fruit stalk. S1: young fruit stage; S2: hard seed stage; S3: coloring and enlargement stage; S4: hard–ripe stage; S5: fully ripe stage.

During the development and maturation of *Cerasus humilis*, the changes in the proportion of different calcium forms in the fruits and fruit stalks under 2,4-D treatment and control (distilled water spraying) are shown in Figure 6. The proportion of water-soluble calcium in the fruits and fruit stalks exhibited an increasing trend, reaching its highest level at the fully ripe stage. The proportion of calcium pectin decreased in the fruits, while the trend in the fruit stalks was less pronounced. The combined proportion of water-soluble calcium and calcium pectin, representing active calcium, showed a gradual increase in the fruits and fruit stalks, peaking at the fully ripe stage. The proportion of calcium oxalate followed a trend of increasing and then decreasing in the fruits and fruit stalks. Calcium phosphate showed a gradual decline in the fruits and fruit stalks. At the fully ripe stage, the proportions of the four main calcium components in the fruits of *Cerasus humilis* differed significantly, following this order: water-soluble calcium > calcium pectin > calcium phosphate > calcium oxalate. Compared to the control, 2,4-D treatment had little effect on the proportions of calcium components in the fruits (Figure 6a,b). The proportion of water-soluble calcium in the 2,4-D-treated fruits was slightly lower than in the control, while the proportions of calcium pectin, calcium phosphate, calcium oxalate, and residual calcium were slightly higher in the 2,4-D-treated fruits (Figure 6a,b). At the fully ripe stage, the proportions of the four main calcium components in the fruit stalks of the control group were nearly equal, with the order being calcium pectin > calcium oxalate > calcium phosphate > water-soluble calcium (Figure 6c). However, in the 2,4-D-treated fruit stalks, significant differences were observed in the proportions of the four calcium components, with calcium pectin and water-soluble calcium comprising a larger proportion, significantly higher than those of calcium oxalate and calcium phosphate (Figure 6d).

In summary, compared to the control, 2,4-D treatment significantly reduced the proportions of calcium oxalate and calcium phosphate in the fruit stalks, while increasing the proportions of calcium phosphate and calcium oxalate in the fruits. This suggests that 2,4-D treatment promotes the transport of the retained calcium phosphate and calcium oxalate from the fruit stalks to the fruits. During the maturation process, 2,4-D treatment had a more pronounced effect on the proportions of calcium in the fruit stalks compared to the control. In contrast, the proportions of calcium in the control fruit stalks and fruits remained relatively stable, with smaller fluctuations (Figure 6).

### 2.4. Relationship Between Calcium Accumulation in Fruits and Calcium in Fruit Stalks of Cerasus humilis

As shown in Table 1, the water-soluble calcium content in the fruit stalks of both *Cerasus humilis* types was highly positively correlated with the water-soluble calcium content in the fruit but exhibited an extremely significant negative correlation with other calcium forms and total calcium. The calcium pectin content in the fruit stalks was highly negatively correlated with the water-soluble calcium content in the fruit and positively correlated with other calcium forms and total calcium to varying degrees. The calcium oxalate content in the fruit stalks showed an extremely significant negative correlation with the water-soluble calcium content in the fruit and a significant or extremely significant positive correlation with other calcium forms and total calcium. The calcium phosphate and active calcium in the fruit stalks also exhibited varying degrees of correlation with the different calcium forms in the fruit (Table 1). These results indicate that the accumulation of calcium nutrients in the fruit is closely linked to the calcium content, variation patterns, and the transport of different calcium forms in the fruit stalks.

As shown in Table 2, the water-soluble calcium content in the fruit stalks under 2,4-D treatment and the control group was significantly positively correlated with the water-soluble calcium content in the fruit and significantly or highly negatively correlated with the content of other calcium forms in the fruit. The calcium pectin content in the fruit stalks was highly negatively correlated with water-soluble calcium in the fruit while positively correlated with other calcium forms. The calcium phosphate content in the fruit stalks showed an extremely significant negative correlation with the water-soluble calcium content in the fruit, with weaker correlations to other calcium forms. The calcium oxalate content in the fruit stalks was highly negatively correlated with water-soluble calcium in the fruit and highly positively correlated with other calcium forms. The total calcium content in the fruit stalks was highly negatively correlated with water-soluble calcium in the fruit while showing significant or extremely significant positive correlations with other calcium forms (Table 2). In summary, the correlations between different calcium forms in the fruit stalks and fruits under 2,4-D treatment and the control group were consistent. However, 2,4-D treatment significantly increased the correlation coefficients between calcium phosphate and calcium oxalate in the fruit stalks and the different calcium forms in the fruit, leading to an overall increase in correlation.

### 2.5. Calcium Concentration Difference Ratio Between the Fruit Stalks and Fruits During Cerasus humilis Fruit Development

As shown in Table 3, during the development and maturation of *Cerasus humilis*, there were significant differences in the content of various calcium forms between the fruits and fruit stalks. The calcium content in the fruit stalks was much higher than in the fruits, exhibiting a clear concentration difference ratio. During fruit development and maturation, the concentration difference ratio of water-soluble calcium showed minor fluctuations but remained relatively stable. The concentration difference ratios of calcium pectin, calcium phosphate, calcium oxalate, active calcium, and total calcium exhibited similar trends, initially decreasing and then increasing. The lowest concentration difference ratio was observed at the hard seed stage, with a range of 4.27–10.06 times, while the highest was at the fully ripe stage, with a range of 14.19–41.30 times, showing an overall increasing trend (Table 3).

In summary, there is a significant difference in calcium content between the fruit stalks and fruits in *Cerasus humilis*. Among the various calcium forms, water-soluble calcium has the smallest concentration difference ratio, making it the form most readily transported from the fruit stalks to the fruits. In contrast, the concentration difference ratios of calcium oxalate and calcium phosphate are the largest, indicating that these forms tend to accumulate in the fruit stalks and are less likely to be transported to the fruits. The behavior of calcium pectin differs between the two *Cerasus humilis* types. In MY-2, the concentration difference ratio of calcium pectin between the fruit stalks and fruits is relatively small, indicating that calcium pectin is easily transported to the fruits. However, in MY-9, the concentration difference ratio of calcium pectin is significantly higher, suggesting that calcium pectin is more difficult to transport to the fruits compared to MY-2 (Table 3).

As shown in Table 4, during the development and maturation of *Cerasus humilis*, there were significant differences in the content of various calcium forms between the fruits and fruit stalks. The calcium content in the fruit stalks was much higher than in the fruits. During fruit development and maturation, the concentration difference ratio of water-soluble calcium showed a gradual decline. The concentration difference ratios of calcium pectin, calcium phosphate, calcium oxalate, active calcium, and total calcium exhibited similar trends, initially decreasing and then increasing. The concentration difference ratio was relatively low at the hard seed stage and reached its highest at the fully ripe stage, showing an overall increasing trend. Except for water-soluble calcium, where the concentration difference ratio was slightly higher in the 2,4-D treatment compared to the control, the concentration difference ratios of other calcium forms were significantly lower in the 2,4-D treatment than in the control (Table 4).

In summary, there is a significant difference in calcium content between the fruit stalks and fruits in *Cerasus humilis*. The 2,4-D treatment markedly reduced the concentration difference ratios of calcium pectin, calcium phosphate, calcium oxalate, active calcium, and total calcium between the fruit stalks and fruits. The reduction in the concentration difference ratio of calcium oxalate was the most notable, suggesting that 2,4-D treatment facilitates the transport of various calcium forms from the fruit stalks to the fruits, thereby increasing the calcium content in the fruits (Table 4).

## 3. Discussion

Calcium is an essential macronutrient for plant growth and development. In the soil, Ca^2+^ is transported to the root surface primarily through mass flow, diffusion, and root interception, before being transferred into the root xylem. From there, it is transported to the aerial parts of the plant via the transpiration stream [29,30]. Fruits, as terminal organs with relatively weak transpiration and independent physiology, receive nutrients from the plant body through the fruit stalk. After calcium is absorbed by the roots, it is transported through the transpiration via the xylem or phloem stream to branches, young leaves, flowers, and fruits. It exists in various calcium forms within these tissues [31,32]. The fruit stalk, serving as the conduit between the tree and the fruit, is the sole pathway through which calcium from the plant body enters the fruit [33,34,35].

The present study found that the changes in the content of different calcium forms in the fruits and fruit stalks of different *Cerasus humilis* types and under 2,4-D treatment followed similar patterns. Both exhibited an overall increase in water-soluble calcium content, while total calcium, active calcium, calcium pectin, calcium phosphate, and calcium oxalate showed general declines, with the decrease being more pronounced in the fruit than in the fruit stalks. It is likely that as the fruit develops and matures, the rapid expansion of fruit cells occurs, with vacuole enlargement being the primary factor driving cell expansion. As an osmotic regulator, calcium accumulates in the vacuoles, primarily in the form of water-soluble calcium. In contrast, the amount of calcium pectin required for cell wall growth is limited. Therefore, during fruit maturation, the accumulation of water-soluble calcium is significantly higher than that of other calcium forms. Compared to distilled water spraying, 2,4-D treatment significantly increased the content of all calcium forms in the fruit, while enhancing the increase in water-soluble calcium content and reducing the decline of calcium pectin, calcium phosphate, calcium oxalate, and total calcium. This effect is likely due to the regulation of calcium absorption, transport, and accumulation by plant hormones. Studies have shown that auxins promote the translocation of calcium from the plant body to the fruit, with IAA and NAA primarily enhancing the symplastic pathway of calcium transport into young fruits [36]. As an auxin analog, 2,4-D not only promotes fruit enlargement and increased fruit weight but also facilitates calcium uptake and accumulation.

During the development and maturation of Cerasus humilis, the proportions of different calcium forms in the fruits and fruit stalks exhibited fluctuating changes, indicating that calcium forms can interconvert during fruit maturation. Previous studies on apples and kiwifruits have demonstrated that calcium can interconvert within the fruit, and the results of this study are consistent with those findings [37,38]. The proportions of the four main calcium components were similar in the fruit stalks, while in the fruits, there were significant differences, with water-soluble calcium and calcium pectin being the dominant forms. At the fully ripe stage, the sum of water-soluble calcium and calcium pectin in the fruits of both *Cerasus humilis* types approached 70%, making them the most prevalent calcium forms in the fruit. The proportion of calcium pectin exhibited clear differences between the two types in the fruits and fruit stalks. In the MY-2 fruits, the proportion of calcium pectin was significantly higher than in the fruit stalks, indicating that calcium pectin is more readily transported from the fruit stalk to the fruit. Conversely, in the MY-9 fruits, the proportion of calcium pectin was lower than in the fruit stalks, suggesting that calcium pectin tends to accumulate in the fruit stalks. This difference could be the primary factor contributing to the variation in calcium content between the two types. The application of 2,4-D altered the distribution of the four major calcium components in the fruit stalks, increasing the proportions of water-soluble calcium and calcium pectin while reducing the proportions of calcium phosphate and calcium oxalate. Furthermore, 2,4-D treatment increased the proportion of calcium phosphate and calcium oxalate in the fruit, suggesting that 2,4-D may promote the transport of these forms from the fruit stalks to the fruits. This effect may be due to hormone regulation of cell expansion, cell wall modification, xylem development, and phloem sucrose unloading. Calcium is known to participate in signaling pathways involving gibberellins, auxins, and abscisic acid, which regulate processes such as fruit set, ripening initiation, cell proliferation, and fruit softening. These physiological changes likely influence calcium distribution within the fruit [39]. The proportions of calcium forms in the fruit stalks and fruits showed clear differences between the young fruit stage and the fully ripe stage, with the most significant changes occurring between the hard–ripe stage and the fully ripe stage. These two stages are critical for calcium changes, transport, and accumulation.

The correlation analysis of calcium nutrition in the fruits and fruit stalks of *Cerasus humilis* reveals that the changes in the content of different calcium forms in the fruits are closely related to those in the fruit stalks. The fruit stalks, as the primary pathway for calcium transport to the fruit, play a crucial role in calcium transport. Studies by Huang et al. [40] found that the calcium concentration in the fruit stalks of lychee is significantly higher than in the fruit. Similarly, Song et al. [41] also found that the calcium content in lychee fruit is an order of magnitude lower than that in their fruit stalks, indicating a significant calcium concentration difference ratio between the fruit stalks and the fruits. Zhong Weiliang [42], using the ^45^Ca isotope, injected calcium into lychee fruit stalks and found that less than 1% of the calcium was transported into the fruits. This study found that during the development and maturation of *Cerasus humilis* fruits, the calcium content in the fruit stalks was much higher than in the fruits, showing a clear concentration difference ratio, suggesting the existence of a ’bottleneck’ in calcium transport between the fruit stalks and the fruits. Calcium in the plant can be divided into two major categories: water-soluble calcium (free Ca^2+^) and non-water-soluble calcium, which includes calcium pectin, calcium phosphate, and calcium oxalate [43]. Calcium pectin, a calcium form associated with the cell wall, plays a crucial role in maintaining its integrity. During fruit growth and softening, calcium pectin undergoes enzymatic degradation, leading to the disintegration of pectin–calcium complexes. The calcium ions released from pectin may either remain as water-soluble calcium or combine with other components to form calcium oxalate and calcium phosphate [44]. Water-soluble calcium and calcium pectin are considered active forms of calcium. Due to the free mobility of water-soluble calcium and the ease with which calcium pectin can be converted, we hypothesize that active calcium (water-soluble calcium and calcium pectin) is more readily translocated and transported within the plant. In contrast, non-active calcium forms, such as calcium oxalate and calcium phosphate, once fixed, are less likely to be redistributed, making them more likely to be retained in the fruit stalks [43,45]. Studies have shown that most of the retained calcium in the fruit stalks is bound to the cell wall or precipitated as calcium oxalate. Electron probe microscopy has revealed large granular calcium oxalate crystals in the phloem of the fruit stalks [46]. Most studies focus on calcium oxalate as the primary cause of the calcium transport “bottleneck”. For instance, Yi Junwen [47] found that the calcium content in longan fruit stalks is higher than in the fruits, with large amounts of calcium oxalate crystals present in the fruit stalks. Zhang Xinsheng et al. [48] found that, in the early stages of apple development, the formation of calcium oxalate in the fruit stalks does not affect calcium influx. However, as the fruit develops, the accumulated calcium oxalate crystals gradually block vascular tissues, hindering later calcium influx. In contrast, Song Wenpei [17] suggested that, in citrus and other fruit trees, calcium retained in the fruit stalks is primarily in the form of calcium oxalate or structural calcium (calcium pectin and calcium phosphate), but the formation of calcium oxalate in the fruit stalks may not be the primary cause of the calcium transport ‘bottleneck’. In this study, different calcium forms exhibited varying characteristics in concentration differences and transport. The concentration difference of water-soluble calcium was the smallest and remained stable, with no significant fluctuations, allowing for easy transport from the fruit stalks to the fruits throughout development. The concentration differences in calcium oxalate and calcium phosphate were the largest, showing only a small difference in the early stages but a significantly larger difference in the later stages. These forms of calcium were more easily transported to the fruits in the early stages but became more likely to remain in the fruit stalks in the later stages, making their transport into the fruits more difficult. Throughout development, the MY-9 fruits exhibited a significantly higher concentration difference compared to MY-2, which may explain the differences in calcium content between the two types. The ’bottleneck’ effect in the MY-9 fruit stalks becomes more pronounced during the later stages of fruit development, with more calcium oxalate and calcium phosphate precipitating in the fruit stalks, leading to reduced calcium transport to the fruits. Further treatment with 2,4-D, an auxin analog, demonstrated that 2,4-D could significantly enhance the correlation between calcium phosphate, calcium oxalate, and the various calcium forms in the fruits. It also notably reduced the concentration differences in the calcium forms between the fruit stalks and fruits, indicating that 2,4-D promotes the transport of non-water-soluble calcium forms, such as calcium pectin, calcium phosphate, and calcium oxalate, from the fruit stalks to the fruits. The application of 2,4-D effectively alleviated the ’bottleneck’ in calcium transport in *Cerasus humilis* fruit. This effect is likely mediated by 2,4-D’s regulation of calcium transporters and channels, which are responsible for calcium uptake and translocation. 2,4-D may enhance the activity of these transporters, thereby increasing the movement of calcium from the fruit stalks to the fruits. However, the precise mechanisms underlying this regulation require further investigation.

## 4. Materials and Methods

### 4.1. Experimental Materials

This experiment utilized two selected types of *Cerasus humilis*: ‘Mengyou No.2’ (MY−2), and ‘Mengyou No.9’ (MY−9). These materials were planted at the *Cerasus humilis* research base of Inner Mongolia Agricultural University, located in Hohhot, Inner Mongolia, at a longitude of 111.65° and a latitude of 40.82°.

Each *Cerasus humilis* plant was maintained with two fruiting branches and six vegetative branches to ensure consistent cultivation practices. During the flowering period (late April), a single foliar application of 0.2% borax solution was administered. At the fruit coloring and swelling stage, two applications of 0.3% monopotassium phosphate solution were applied. Irrigation was managed according to soil moisture conditions, with watering during key stages: the budding phase (beginning in early April), the fruit swelling phase (starting in early August), and before soil freeze. Watering was restricted during the flowering period, and irrigation ceased 7 to 10 days before fruit maturity.

### 4.2. Sample Collection

The experiment was conducted between June and September 2022, using Cerasus humilis MY-2 and MY-9 as experimental materials. The flowering date was recorded as April 24. Samples were collected at five stages: 50 days post-flowering on June 14 (young fruit stage, S1), 80 days post-flowering on July 14 (hard seed stage, S2), 120 days post-flowering on August 24 (coloring and enlargement stage, S3), 135 days post-flowering on September 10 (hard–ripe stage, S4), and 150 days post-flowering on September 25 (fully ripe stage, S5). A total of 300 fruits and their corresponding fruit stalk samples were collected from the two types of *Cerasus humilis*, ensuring uniformity, the absence of pests and diseases, and no mechanical damage. For each sampling period, five fruits were selected from the upper, middle, and lower positions of each fruiting branch across 10 plants, resulting in a total of 20 fruiting branches. The collected fruits and fruit stalks were mixed uniformly and divided into three portions. After washing with distilled water and air-drying at room temperature, the samples were rapidly frozen in liquid nitrogen and stored in a −80 °C freezer until all samples were collected for subsequent data analysis.

From May to September 2023, *Cerasus humilis* MY-9 was used as the experimental material. The flowering date was recorded as April 20. Ten uniformly growing *Cerasus humilis* trees were selected, and during the peak calcium formation period, a 2,4-D solution was applied four times: on May 10, May 17, July 15, and July 22. Using an electric sprayer, 1L of a 10 *ppm* 2,4-D solution was accurately sprayed onto each fruiting branch, with distilled water sprayed onto the fruiting branches of 10 other uniform trees as a control. Samples were collected at five stages: 50 days post-flowering on June 10 (young fruit stage, S1), 80 days post-flowering on July 10 (hard seed stage, S2), 120 days post-flowering on August 20 (coloring and enlargement stage, S3), 135 days post-flowering on September 5 (hard–ripe stage, S4), and 150 days post-flowering on September 20 (fully ripe stage, S5). A total of 300 fruit and fruit stalk samples were collected from the *Cerasus humilis* under both the 2,4-D treatment and the control (plain water spray), ensuring uniformity, the absence of pests and diseases, and no mechanical damage. For each sampling period, five fruits were selected from the upper, middle, and lower positions of each fruiting branch across 10 plants, resulting in a total of 20 fruiting branches. The collected fruits and fruit stalks were mixed uniformly and divided into three portions. After washing with distilled water and air-drying at room temperature, the samples were rapidly frozen in liquid nitrogen and stored in a −80 °C freezer until all samples were collected for subsequent data analysis.

### 4.3. Experimental Index Measurement

The extraction of different forms of calcium was conducted according to the method described by Huang et al. [49]. Samples of 1.0 g of *Cerasus humilis* at different developmental stages were sequentially extracted using ultra-pure water, 1 mol/L sodium chloride, 2% acetic acid, and 5% hydrochloric acid in a water bath at 25 °C. The calcium extracted with ultra-pure water was classified as water-soluble calcium, the calcium extracted with sodium chloride was identified as calcium pectin, the calcium extracted with acetic acid was classified as calcium phosphate, and the calcium extracted with hydrochloric acid was identified as calcium oxalate. Finally, the remaining residue was digested using a mixed acid of HNO₃-HClO₄ (5:1) to determine the residual calcium content. The concentrations of the various forms of calcium were measured using a flame atomic absorption spectrophotometer (Hitachi ZA3000 series, Hitachi Scientific Instruments Co., Ltd., Beijing, China). Each measurement was repeated three times.

Active calcium = Water-soluble calcium + Calcium pectin(1)

Total calcium = Water-soluble calcium + Calcium pectin + Calcium phosphate + Calcium oxalate + Residual calcium(2)

### 4.4. Statistical Analysis

In the data analysis, Microsoft Excel 2010 and GraphPad Prism 8.0.2 were utilized for data processing and the creation of bar charts, while SPSS 25.0 was employed for statistical analysis and Pearson’s correlation analysis. The significance of differences was examined using ANOVA analysis and Duncan’s multiple comparisons with a significance level of *p* < 0.05.

## 5. Conclusions

In summary, during the development and maturation of *Cerasus humilis* fruit, the content and proportion of different calcium forms in the fruit and fruit stalk exhibited similar patterns and were closely related. Compared to the fruit stalks, the mature fruit contains a higher proportion of active calcium and water-soluble calcium, while the proportions of calcium phosphate and calcium oxalate are lower. A significant calcium concentration difference ratio existed between the fruit stalks and the fruits, with the smallest ratio observed for water-soluble calcium, the largest for calcium phosphate and calcium oxalate, and an intermediate ratio for calcium pectin. Water-soluble calcium is efficiently transported from the fruit stalks to the fruits, while calcium phosphate and calcium oxalate tend to remain in the fruit stalks. The calcium pectin transport capacity differed between the two types: MY-2 exhibited stronger transport to the fruits, while MY-9 showed a greater tendency for calcium pectin accumulation in the fruit stalks. The critical stages for calcium changes and accumulation occurred from the young fruit stage to the hard seed stage and from the hard–ripe stage to the fully ripe stage. Treatment with 2,4-D significantly reduced the content and proportion of calcium oxalate and calcium phosphate in the fruit stalks, while increasing the content and proportion of various calcium forms in the fruits. This resulted in a decreased calcium concentration difference ratio between the fruit stalks and the fruits, thereby promoting calcium transport into the fruits. The application of 2,4-D as a plant growth regulator provides a scientific basis for further investigation into the calcium metabolism of *Cerasus humilis* and the optimization of calcium regulation. Moreover, it offers a new perspective for enhancing fruit quality and refining calcium application strategies. However, this study is based on data from a single year and offers a preliminary exploration of the calcium transport relationship between the fruit stalks and fruits, as well as the effect of 2,4-D on calcium transport in fruits. Future research should encompass multiple fruit species and involve multi-year replicates to validate the dynamic accumulation patterns and transport characteristics of calcium in the fruit stalks and fruits.

## Figures and Tables

**Table 1:** The correlation between fruit calcium content and fruit stalk calcium content in *Cerasus humilis*.

Types	Fruit Calcium Fractions	Fruit Stalk Calcium Fractions					
		Water-Soluble Calcium	Calcium Pectin	Calcium Phosphate	Calcium Oxalate	Active Calcium	Total Calcium
MY−2	Water-soluble calcium	0.882 **	−0.672 **	−0.429	−0.843 **	−0.021	−0.478
	Calcium pectin	−0.699 **	0.604 *	0.309	0.703 **	0.087	0.414
	Calcium phosphate	−0.848 **	0.682 **	0.384	0.824 **	0.056	0.472
	Calcium oxalate	−0.750 **	0.435	0.133	0.590 *	−0.116	0.229
	Active calcium	−0.667 **	0.585 *	0.291	0.674 **	0.092	0.400
	Total calcium	−0.782 **	0.610 *	0.302	0.738 **	0.033	0.404
MY−9	Water-soluble calcium	0.946 **	−0.847 **	−0.670 **	−0.717 **	−0.201	−0.615 *
	Calcium pectin	−0.828 **	0.722 **	0.412	0.584 *	0.148	0.449
	Calcium phosphate	−0.910 **	0.884 **	0.683 **	0.762 **	0.289	0.669 **
	Calcium oxalate	−0.853 **	0.630 *	0.340	0.502	−0.005	0.335
	Active calcium	−0.811 **	0.704 **	0.387	0.568 *	0.142	0.431
	Total calcium	−0.881 **	0.777 **	0.501	0.643 **	0.171	0.513

Note: “*” indicates a significant correlation at the *p* < 0.05 level, and “**” indicates an extremely significant correlation at the *p* < 0.01 level.

**Table 2:** The correlation between fruit calcium content and fruit stalk calcium content in *Cerasus humilis* under 2,4-D treatment.

Treatments	Fruit Calcium Fractions	Fruit Stalk Calcium Fractions					
		Water-Soluble Calcium	Calcium Pectin	Calcium Phosphate	Calcium Oxalate	Active Calcium	Total Calcium
CK	Water-soluble calcium	0.892 **	−0.726 **	−0.762 **	−0.907 **	−0.063	−0.810 **
	Calcium pectin	−0.810 **	0.745 **	0.265	0.849 **	0.172	0.576 *
	Calcium phosphate	−0.957 **	0.783 **	0.555 *	0.970 **	0.072	0.739 **
	Calcium oxalate	−0.883 **	0.609 *	0.211	0.865 **	−0.086	0.465
	Active calcium	−0.765 **	0.716 **	0.191	0.806 **	0.179	0.522 *
	Total calcium	−0.886 *	0.743 **	0.338	0.906 **	0.091	0.609 *
2,4−D	Water-soluble calcium	0.911 **	0.101	−0.820 **	−0.754 **	0.830 **	−0.689 *
	Calcium pectin	−0.765 **	0.619 *	0.319	0.978 **	−0.259	0.657 **
	Calcium phosphate	−0.885 **	0.368	0.669 **	0.967 **	−0.516 *	0.833 **
	Calcium oxalate	−0.755 **	0.661 **	0.229	0.939 **	−0.224	0.573 *
	Active calcium	−0.700 **	0.691 **	0.226	0.955 **	−0.159	0.615 *
	Total calcium	−0.800 **	0.594 *	0.386	0.988 **	−0.303	0.701 **

Note: “*” indicates a significant correlation at the *p* < 0.05 level, and “**” indicates an extremely significant correlation at the *p* < 0.01 level.

**Table 3:** The calcium concentration difference ratio in fruit stalk and fruit during the development and ripening of *Cerasus humilis*.

Types	Fruit Development Stage	Fruit Stalk Calcium Concentration/Fruit Calcium Concentration					
		Water-Soluble Calcium	Calcium Pectin	Calcium Phosphate	Calcium Oxalate	Active Calcium	Total Calcium
MY-2	S1	12.22	8.02	7.42	24.37	9.04	10.32
	S2	11.32	4.32	5.41	10.06	5.39	6.33
	S3	13.89	6.54	9.09	17.18	8.07	9.86
	S4	12.71	6.58	10.01	20.25	8.20	10.40
	S5	12.65	15.81	34.13	37.50	14.19	20.76
MY-9	S1	12.14	7.83	7.12	20.29	8.87	9.75
	S2	11.64	4.27	5.47	9.42	5.31	6.17
	S3	13.39	6.95	8.38	18.68	8.49	9.95
	S4	12.87	7.22	10.13	21.56	8.86	10.97
	S5	12.52	29.57	39.19	41.30	18.03	25.24

**Table 4:** The calcium concentration difference ratio in fruit stalk and fruit during the development and ripening of *Cerasus humilis* under 2,4-D treatment.

Treatments	Fruit Development Stage	Fruit Stalk Calcium Concentration/Fruit Calcium Concentration					
		Water-Soluble Calcium	Calcium Pectin	Calcium Phosphate	Calcium Oxalate	Active Calcium	Total Calcium
CK	S1	14.64	7.74	7.56	19.39	9.26	10.10
	S2	12.56	4.42	4.50	11.24	5.63	6.32
	S3	13.55	7.29	7.93	20.29	8.74	10.10
	S4	13.48	7.14	9.79	22.97	9.00	11.00
	S5	13.02	25.90	25.07	39.99	17.39	21.17
2,4-D	S1	18.46	6.19	6.38	13.99	9.12	8.86
	S2	16.19	3.69	3.87	7.32	5.62	5.53
	S3	15.24	5.37	5.33	10.00	7.72	7.52
	S4	14.39	6.57	6.49	13.11	9.00	9.13
	S5	13.42	20.60	15.02	23.53	16.16	17.23

## Data Availability

The data are contained within this article.
